# Evaluation of Dielectric Endurance of Nano-Additive Reinforced Polyester Composites via Hankel-RPCA Decomposition

**DOI:** 10.3390/polym18080992

**Published:** 2026-04-19

**Authors:** Mete Pınarbaşı, Fatih Atalar, Aysel Ersoy

**Affiliations:** Department of Electrical and Electronic Engineering, Istanbul University-Cerrahpasa, University St. No. 7, Avcılar, 34320 Istanbul, Turkey; metepinarbasi@hotmail.com (M.P.); aersoy@iuc.edu.tr (A.E.)

**Keywords:** nano material, dielectric endurance, Robust Principal Component Analysis (RPCA), hankel matrix, polymer

## Abstract

Surface discharge-induced degradation poses a significant threat to the operational reliability of high-voltage insulation systems. This research investigates the dielectric endurance of polyester-based nanocomposites reinforced with seven distinct nano-additives: iron oxide (Fe_3_O_4_), copper oxide (CuO), titanium oxide (TiO_2_), aluminum oxide (Al_2_O_3_), silicon dioxide (SiO_2_), zinc borate (ZnB) and graphene oxide (GO). Specimens were fabricated at 0.5% and 0.75% weight concentrations and subjected to constant AC electrical stress of 4.5 kV at 50 Hz until failure using the first-plane tracking method. To accurately monitor the aging process, a sophisticated signal processing framework involving Hankel-matrix-enhanced Robust Principal Component Analysis (RPCA) was developed to extract high-frequency discharge features from captured leakage current signals. The degradation characteristics were quantified using various statistical metrics, including Kurtosis, RMS and Burst Discharge Index (BDI). Experimental findings demonstrate that the incorporation of nanoparticles significantly extends the time-to-failure compared to neat polyester, although the effectiveness is highly dependent on both additive type and concentration. At 0.5 wt.%, ZnB exhibited the superior performance in delaying carbonized track formation. However, at 0.75 wt.%, Al_2_O_3_ emerged as the most effective additive, achieving a maximum endurance time of 31.61 min. In contrast, certain additives like TiO_2_ showed a performance decline at higher loadings, likely due to nanoparticle agglomeration. The Hankel-RPCA methodology successfully isolated discharge-specific signatures from background noise, establishing a strong correlation between signal features and material failure stages. This study confirms that the synergy between advanced nanomaterial modification and robust signal processing provides an effective diagnostic tool for monitoring insulation health, offering a vital pathway for the designing of high-performance dielectrics for real-world power system applications.

## 1. Introduction

Polymeric insulating materials underpin modern electrical infrastructure, favored for their low density, flexibility, and intrinsically high dielectric strength. While essential for electrical machines, power cables and electronic devices, their reliability under sustained AC stress is frequently compromised by surface degradation phenomena. Specifically, leakage current escalation and surface discharge activity dictate long-term dielectric failure processes [1]. Consequently, enhancing surface discharge resistance and extending dielectric lifetime remain critical imperatives in insulation research.

A primary strategy to overcome these limitations is the engineering of the polymer matrix via inorganic nano-additives. The incorporation of nanofillers such as Al_2_O_3_, SiO_2_, TiO_2_, zinc borate (ZnB) and graphene oxide (GO) generates extensive interfacial regions that fundamentally alter charge transport, trap distributions and electric field homogeneity at the nanoscale [2,3,4]. Research indicates that these interfacial mechanisms correlate with increased dielectric breakdown strength and reduced leakage current density, provided the dispersion remains homogeneous [5,6]. For instance, the embedding of Al_2_O_3_ nanosheets has been shown to improve breakdown strength by approximately 25% through the suppression of leakage pathways and modification of local field distributions [7]. Similarly, Thabet et al. demonstrated that optimized nano-silica loadings significantly bolster surface discharge resistance by homogenizing charge pathways [8]. However, this enhancement is sensitive; excessive or poorly dispersed nanofillers can act as defect sites, causing localized field enhancement that acts to deteriorate, rather than improve, insulation performance [3,9].

The observed concentration-dependent performance of the nanocomposites is further elucidated by considering the microscopic dynamics within the polymer matrix. As demonstrated in recent broadband dielectric studies on polyurethane and polyesterimide nanocomposites, the incorporation of nanoparticles such as Al_2_O_3_ and ZnO can fundamentally alter polymer-chain mobility and relaxation behavior. Specifically, these fillers can either restrict segmental motion through strong polymer-filler binding or facilitate the formation of looser interfacial structures, depending on the matrix type and filler loading. Such modifications in chain dynamics and local permittivity provide a vital mechanistic context for the shift in discharge patterns and the resulting variations in macroscopic dielectric endurance observed in this study [10,11].

While traditional static metrics like breakdown strength and leakage current magnitude offer baseline insights, they fail to quantify the dynamic micro discharge activities driving degradation. Under AC stress, leakage current waveforms comprise both baseline conduction and high-frequency transient components indicative of partial discharges (PD) and surface discharge events, features often obscured in averaged metrics [12]. Although conventional spectral methods, such as Fourier transform or wavelet analysis, can capture frequency content, they struggle to effectively decouple overlapping baseline and transient features within short, non-stationary recordings [13,14]. Recent reviews highlight that while advanced techniques like continuous wavelet transform (CWT), empirical mode decomposition (EMD) and artificial neural networks have been employed for fault diagnosis, a standardized framework for isolating physically meaningful discharge signatures remains elusive [15,16].

Robust Principal Component Analysis (RPCA) presents a transformative alternative by decomposing data matrices into a low-rank component (regular conduction) and a sparse component (transient discharge pulses) [17]. When augmented with Hankel matrix embedding, RPCA facilitates the physically interpretable separation of baseline leakage from sparse discharge signatures, even amidst significant noise [18,19]. While such decomposition strategies have proven superior to traditional PCA and wavelet approaches in mechanical and electrical anomaly detection [20,21], their application to leakage current signals in polymer nanocomposites remains largely unexplored. This creates a notable gap in our ability to correlate specific signal features with material modifications. Critically, while existing literature acknowledges that nano-additives can attenuate high-frequency noise or modify discharge patterns, there is a distinct lack of quantitative linkage between these microscopic behaviors and macroscopic endurance metrics, such as voltage withstand capability or time-to-failure [22,23,24]. Bridging this divide requires a methodology that not only extracts discharge-related features but also correlates them directly with mechanical lifetime outcomes.

To address these limitations, the present study introduces an integrated experimental and signal-processing framework that directly links nano-additive modification to surface discharge dynamics and dielectric endurance performance. In this study, in order to systematically investigate the structure-property relationships governing dielectric endurance, seven distinct nano-fillers were strategically selected based on their diverse physical and chemical mechanisms for reinforcing the polyester matrix. Metal oxides, specifically Al_2_O_3_, SiO_2_ and TiO_2_, were chosen as archetypal inorganic reinforcements known for establishing robust interfacial barriers that obstruct leakage current pathways. These fillers, particularly Al_2_O_3_ and SiO_2_ facilitate the formation of a thermally stable, erosion-resistant layer, which is vital for mitigating surface tracking and dissipating localized thermal energy under sustained AC stress. TiO_2_ was specifically included to evaluate the role of high-permittivity fillers in modifying local electric field distributions and grading the stress at the polymer-filler interface. To explore the influence of electronic and magnetic functionalities on discharge dynamics, semiconductive and magnetic nanoparticles Fe_3_O_4_ and CuO were incorporated. These additives serve to investigate how magnetic permeability and narrow band-gap characteristics interfere with electron avalanche development and surface charge accumulation. Furthermore, Zinc Borate (ZnB) was selected as a multifunctional additive for its unique flame-retardant properties and its proven efficacy in promoting the formation of a non-conductive, ceramic-like char layer, which is critical for suppressing carbonization during high-temperature discharge events. Lastly, Graphene Oxide (GO) was chosen to assess the impact of two-dimensional, high-surface-area carbonaceous structures on the creation of a high density of deep charge traps within the polymer interphase, aimed at immobilizing free carriers and enhancing the overall dielectric integrity.

Pure organic polyester was modified with seven different nano-additives incorporated at two controlled low loading levels of 0.5 wt.% and 0.75 wt.% to systematically investigate interfacial effects while avoiding filler agglomeration. High-frequency leakage current signals recorded during surface discharge testing were analyzed using Hankel matrix embedding combined with Robust Principal Component Analysis (RPCA), enabling the decomposition of the signals into low-rank baseline conduction and sparse discharge components. Discharge-related metrics, including high-frequency energy content and burst characteristics extracted from the sparse component, were subsequently correlated with endurance time to quantitatively assess the influence of nano-additive type and concentration. By establishing a direct relationship between nano-additive-induced modifications in sparse discharge behavior and macroscopic dielectric endurance, this study provides a physically interpretable and quantitative framework for the design and optimization of polymer nanocomposite insulation systems beyond conventional dielectric characterization approaches.

## 2. Materials and Methods

### 2.1. Material Preparation and Nanocomposite Fabrication

**Figure 1:**
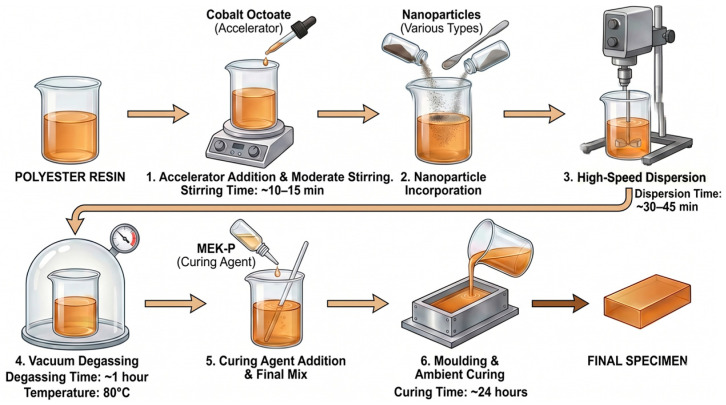
Fabrication steps for nano-additive reinforced polyester samples.

The fabrication of all specimens was carried out under strictly controlled laboratory conditions to ensure homogeneity, repeatability, and statistical reliability. The overall sample preparation workflow is illustrated in Figure 1. A general-purpose unsaturated polyester resin was selected as the base polymer matrix due to its widespread use in electrical insulation systems and well-established surface degradation behavior under AC stress.

Prior to nanoparticle incorporation, the polyester resin (procured from local commercial sources) was conditioned at room temperature to ensure consistent viscosity. Cobalt octoate (procured from local commercial sources) was added to the resin at a concentration of 0.2 wt.% as an accelerator to promote room-temperature curing. The mixture was mechanically stirred at moderate speed until a visually uniform solution was obtained [25]. Following the addition of the MEK-P curing agent (procured from local commercial sources), the mixture was poured into the molds and allowed to cure at ambient temperature (23±2 °C) for 24 h. All samples were then stored in a controlled environment for an additional 48 h to ensure complete chemical stabilization prior to electrical testing.

Inorganic nano-additives, iron oxide (Fe_3_O_4_), copper oxide (CuO), titanium oxide (TiO_2_), aluminum oxide (Al_2_O_3_), silicon dioxide (SiO_2_), zinc borate (ZnB) and graphene oxide (GO), were introduced into the resin at two controlled loading levels of 0.5 wt.% and 0.75 wt.%. The details about the nanofillers are summarized in Table 1. These low filler concentrations were deliberately selected to enhance interfacial polarization and charge trapping effects while minimizing the risk of particle agglomeration and viscosity-induced processing defects. To ensure the homogeneous dispersion of nanoparticles within the polyester matrix, a multi-stage optimization procedure was conducted prior to final specimen fabrication. This protocol was developed based on established preparation workflows from prior research, where similar high-shear mixing and degassing steps were validated for improving the dielectric properties of polymer nanocomposites [26]. In this study, the nanofillers were introduced at controlled low loading levels (0.5% and 0.75 wt.%.) specifically to mitigate the risk of particle agglomeration. The dispersion quality was qualitatively ensured through iterative trial mixtures where visual uniformity and batch-to-batch reproducibility were evaluated. The final standardized workflow involved high-speed mechanical stirring (DLAB OS70-Pro) at 1000 rpm for 30–45 min to disrupt initial clusters, followed by vacuum (Thermomac VO24) degassing for 1 h at the 80 °C to eliminate entrapped air and prevent void-induced field distortions. To verify the consistency of this optimized procedure, five independent specimens were fabricated and tested for each formulation, demonstrating high statistical reliability in the resulting dielectric endurance data [27]. All specimens were cured at ambient temperature under identical conditions. Neat polyester samples were fabricated using the same protocol without nanoparticle addition to serve as reference materials.

### 2.2. Inclined Plane Test (IPT) and Signal Acquisition

**Figure 2:**
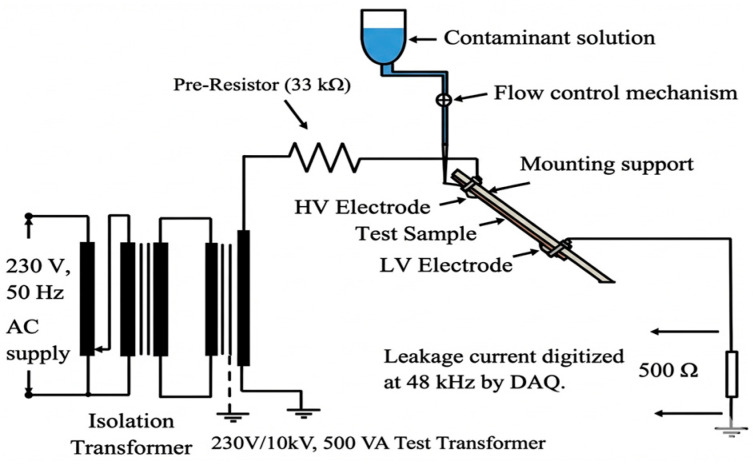
Inclined Plane Test Setup Configuration.

The surface electrical endurance of the specimens was evaluated using the Inclined Plane Test (IPT) strictly in accordance with the IEC 60587 standard [28]. The setup consisted of electrodes mounted on the specimen surface at a 45° angle. A standard contaminant solution (ammonium chloride with a wetting agent) flowed continuously over the surface to simulate polluted environmental conditions as shown in Figure 2. The specimens were cast into rectangular molds with dimensions of 120 mm × 50 mm × 6 mm in strict accordance with the IEC 60587 standard [28]. To ensure the statistical significance of the dielectric endurance data, five independent measurements were performed for each composite formulation and the average values were reported.

A sinusoidal AC voltage 50 Hz was applied at a constant severity level of 4.5 kV by using Esitaş brand test transformer with 230V/10 kV turn ratio. The test proceeded until the failure criterion was met, defined either by the formation of a conductive tracking path bridging the electrodes or by the leakage current exceeding the standard threshold of 60 mA for 2 s.

Simultaneously, leakage current signals were continuously digitized using a high-speed data acquisition unit (DAQ) developed in-house at the Department of Electrical and Electronic Engineering, Istanbul University-Cerrahpaşa at a sampling rate of 48 kHz. This high temporal resolution was chosen to satisfy the Nyquist criterion for capturing broad-band transient discharge events, extending well beyond the fundamental power frequency. For each specimen, the “endurance time” which is defined as the duration from voltage application to failure, was recorded as the primary macroscopic performance metric.

**Figure 3:**
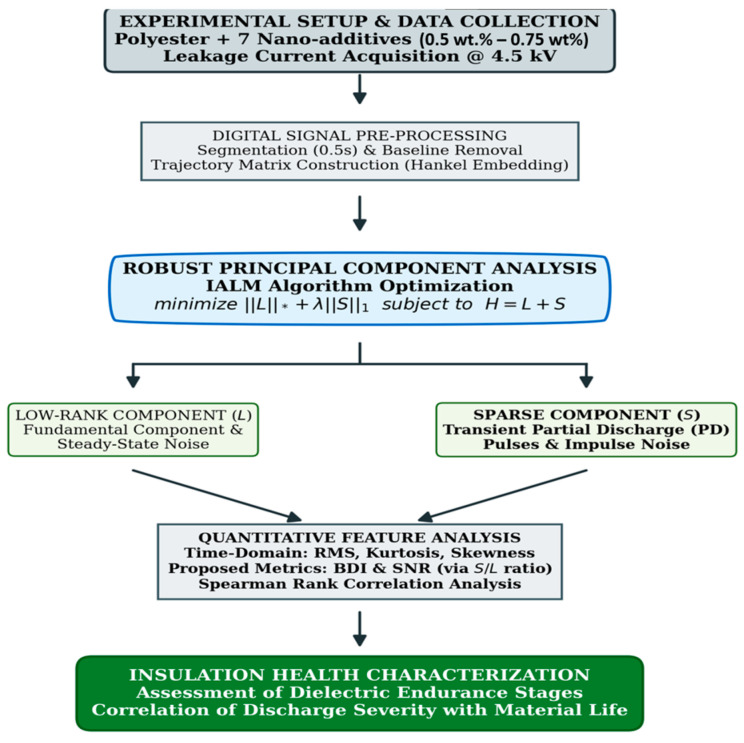
Systematic representation of the insulation health assessment methodology based on Robust Principal Component Analysis (RPCA). Note: $|L{|}_{*}$ denotes the nuclear norm of the low-rank matrix L.

From the continuous data stream, quasi-stationary windows of 0.5 s, exhibiting characteristic discharge bursts, were extracted for post-processing. Segments were manually selected from steady-state regions of the leakage current signal, avoiding switching transients and acquisition artifacts. Preprocessing steps included DC-offset removal and amplitude normalization to ensure numerical stability during subsequent matrix operations. The complete experimental and signal processing framework is summarized in Figure 3.

### 2.3. Hankel Matrix Embedding

To transition from a one-dimensional time-series analysis to a subspace-based decomposition framework, the preprocessed leakage current segments were mapped into a trajectory matrix using Hankel embedding. This transformation is pivotal as it converts the linear dynamical properties of the univariate time series into the algebraic structure of a matrix, thereby revealing hidden correlations between lagged signal components [29].

For a discrete-time leakage current signal vector $x={\left[x_{1},x_{2},\ldots ,x_{N}\right]}^{T}$ of length N, the corresponding Hankel matrix $H\in R^{m\times n}$ is constructed by sliding a window of length m over the data. The matrix is defined such that the $\left(i,j\right)-th$ entry is given by $H_{i,j}=x_{i+j-1}$. The resulting structure is [30]:

$$H=H\left(x\right)=\left[\begin{array}{cccc} x_{1} & x_{2} & \cdots & x_{n}\\ x_{2} & x_{3} & \cdots & x_{n+1}\\ \vdots & \vdots & \ddots & \vdots \\ x_{m} & x_{m+1} & \cdots & x_{N} \end{array}\right]$$

where the embedding dimension m and the number of columns n satisfy the condition m+n−1=N. In this study, m was selected to be approximately N/2 to maximize the separability of the signal components, creating a near-square matrix structure that enhances the singular value spectrum’s discriminative power.

Within this high-dimensional embedding, the signal components manifest distinct algebraic ranks. The baseline leakage current, driven by the 50 Hz power frequency and low-order harmonics, forms a highly correlated, redundant structure corresponding to a low-rank subspace. Conversely, the partial discharge (PD) pulses, which are stochastically modulated by the nanoparticle interfaces, appear as transient, high-frequency events. These events disrupt the linear correlation of the trajectory matrix, manifesting as sparse outliers [31]. This rank-sparsity duality provides the theoretical foundation for isolating the discharge activity specifically influenced by the material’s nanostructure.

### 2.4. Robust Principal Component Analysis (RPCA)

Standard Principal Component Analysis (PCA) is often insufficient for leakage current analysis because it relies on the assumption of Gaussian noise and is sensitive to gross corruptions, such as high-magnitude discharge impulses. To address this, Robust Principal Component Analysis (RPCA) via Principal Component Pursuit (PCP) was employed to robustly decouple the discharge events from the background conduction [32].

The Hankel matrix H is modeled as the superposition of a low-rank component L, representing the quasi-stationary background current and a sparse component S, capturing the transient discharge events:

H=L+S

The mathematical challenge lies in recovering both L and S simultaneously. This is achieved by solving a convex optimization problem that minimizes the nuclear norm of L (surrogate for rank) and the $l_{1}-norm$ of S (surrogate for sparsity) [33]:

$$\underset{L,S}{\min}{\left|L\right|}_{*}+\lambda |S{|}_{1}\;\mathrm{subject}\;\mathrm{to}\;H=L+S$$

Here, $|L{|}_{*}=\sum_{i}\sigma_{i}\left(L\right)$ ensures the background signal remains low-dimensional, while $|S{|}_{1}=\sum_{ij}\left|S_{ij}\right|$ promotes sparsity, ensuring that the extracted component contains only significant discharge events. The regularization parameter λ balances these two terms and is set theoretically as $\lambda =1/\sqrt{\max\left(m,n\right)}$. Default parameter settings commonly used in signal separation literature were employed.

This decomposition is particularly relevant for nanocomposite insulation research. By isolating the sparse component S, it can be effectively filtered out the macroscopic conduction current and focus exclusively on the micro-discharge dynamics, which are the primary indicators of how nanofillers alter the local electric field distribution and suppress electron avalanches [34].

### 2.5. Feature Extraction and Statistical Analysis

Following the decomposition, the sparse matrix S was reconstructed into a one-dimensional time series to enable feature extraction. Since the sparse component represents the isolated discharge activity stripped of the power-frequency background, it serves as a direct proxy for the material’s resistance to surface degradation. A set of physics-informed statistical features was extracted to quantify how different nano-additives modify the discharge patterns [35].

Given the non-Gaussian and impulsive nature of surface discharges, higher-order statistics were prioritized. Kurtosis Ku was calculated to measure the “peakedness” of the discharge distribution. A reduction in kurtosis in nanocomposites compared to the neat polymer typically indicates a suppression of high-intensity discharge bursts, attributed to the scattering effects of nanoparticles. Kurtosis is defined as [36]:

$$Ku=\frac{1}{N}\sum_{i=1}^{N}{\left(\frac{s_{i}-\mu }{\sigma }\right)}^{4}$$

where $s_{i}$ represents the discrete samples of the sparse signal, μ is the mean, and σ is the standard deviation.

To capture the spectral signature of the degradation, the High-Frequency Energy Ratio (HFER) was computed. This metric quantifies the proportion of energy dissipated in the discharge frequency bands (>500 Hz) relative to the total signal energy, reflecting the intensity of ionization processes [37]:

$$\mathrm{HFER}=\frac{\int_{f_{1}}^{f_{2}}{\left|X\left(f\right)\right|}^{2}\;df}{\int_{0}^{f_{max}}{\left|X\left(f\right)\right|}^{2}\;df}$$

Finally, to link these microscopic signal features to macroscopic performance, Spearman’s Rank Correlation Coefficient (ρ) was employed. Unlike linear Pearson correlation, Spearman’s method assesses monotonic relationships, making it robust for linking stochastic discharge features with the non-linear aging behavior of insulation materials [38]. The correlation is given by:

$$\rho =1-\frac{6\sum d_{i}^{2}}{n\left(n^{2}-1\right)}$$

where $d_{i}$ is the rank difference between the signal feature and the recorded endurance time. This statistical framework allows for the identification of specific discharge characteristics that serve as reliable precursors to dielectric failure.

## 3. Results

### 3.1. Statistical Characteristics of Leakage Current Signals

The statistical characteristics of the leakage current signals obtained from nanoparticle-reinforced polyester insulation systems were evaluated using several signal features extracted from the manually selected 0.5 s signal segments. The selection of the 0.5 s signal segments was performed based on a physically guided criterion rather than arbitrary manual choice. Specifically, segments were extracted from quasi-stationary regions following the onset of surface degradation, where repetitive discharge bursts were observed. These regions correspond to a stable pre-failure regime of the leakage current signal, avoiding both initial transients and final breakdown conditions. Although the signals were normalized for processing, the selected segments consistently represent comparable discharge conditions across all samples, ensuring that the extracted features are not biased by isolated or extreme events. The analyzed parameters included root mean square (RMS), crest factor, kurtosis, zero-crossing rate (ZCR), Teager energy operator response, burst discharge index (BDI), transient impulsiveness factor (TIF) and high-frequency spectral ratio (HFSR). A conservative threshold (5% of the normalized PSD) was selected to identify dominant spectral regions while avoiding noise-driven peaks. In addition, the electrical endurance time of each material was recorded and incorporated into the dataset to allow direct comparison between discharge behavior and insulation lifetime. All signal processing, decomposition algorithms (Hankel-RPCA), and statistical analyses were implemented using Python (version 3.14.2). The complete feature sets for the two nanoparticle loading ratios are presented in Table 2 and Table 3. Table 2 summarizes the results for the 0.5 wt.% nanoparticle concentration.

Significant variations are observed among the investigated nanoparticle systems. The endurance time varies from 6.85 min to 72.36 min, indicating a wide range of electrical stability across the tested materials. The lowest endurance time is obtained for the Al_2_O_3_ system (6.85 min), followed by CuO (7.96 min), while the highest endurance time is observed for ZnB (72.36 min). The neat polyester reference exhibits an endurance time of 12.46 min, which lies within the lower portion of the endurance range. The RMS values remain relatively close for most nanoparticle systems, ranging between 0.015 and 0.021, indicating that the overall leakage current magnitude does not vary drastically between materials at this concentration level. However, more pronounced differences appear in the impulsive characteristics of the signals.

The crest factor values show large variations across materials, ranging from 3.56 for the neat polyester sample to 17.18 for the Al_2_O_3_ system. Such high crest factors indicate the presence of strong transient peaks within the leakage current waveform. Similarly, kurtosis values vary considerably among the materials, reaching values as high as 31.93 for Fe_3_O_4_, which suggests the presence of highly intermittent discharge bursts within the signal.

The Teager energy operator values also exhibit notable differences. While the neat polyester reference presents a very low value of approximately 6.02 × 10^−6^, the nanoparticle systems show significantly higher values, reaching 1.59 × 10^−4^ for ZnB, indicating stronger instantaneous energy variations in the leakage current waveform.

The burst discharge index (BDI) values range between 0.0177 and 0.0280, with the highest value observed for the GO system (0.0280). In addition, the transient impulsiveness factor (TIF) varies between 1.38 and 5.82, with the largest value obtained for Fe_3_O_4_ (5.82), indicating pronounced transient discharge behaviour in this system.

The high-frequency spectral ratio (HFSR) values also exhibit noticeable variation. The lowest value is observed for the neat polyester reference (0.0136), whereas significantly higher values are observed for nanoparticle systems, reaching 0.1413 for Fe_3_O_4_ and 0.1373 for CuO. These results indicate that the nanoparticle systems generally contain stronger high-frequency spectral components within the leakage current signals. The results for the higher nanoparticle loading ratio are presented in Table 3.

At the 0.75 wt.% nanoparticle concentration, the statistical characteristics of the leakage current signals exhibited similar trends, although some changes in discharge behavior were observed. The neat polyester reference maintained an endurance time of 12.46 min, while several nanoparticle systems demonstrated noticeable deviations from this baseline. At this concentration level, the endurance times range between 9.44 min and 31.61 min. The longest endurance time is observed for Al_2_O_3_ (31.61 min), followed by ZnB (22.17 min), while the lowest endurance time is obtained for TiO_2_ (9.44 min). Compared with the 0.5 wt.% dataset, the RMS values exhibit a wider range, extending from 0.015 to 0.491, indicating noticeable differences in the magnitude of the leakage current signals among the investigated systems.

The crest factor values are significantly lower than those observed in the 0.5 wt.% dataset, ranging between 2.03 and 4.88, suggesting that the amplitude distribution of the leakage current signals becomes less impulsive at the higher nanoparticle concentration. Similarly, kurtosis values decrease considerably and even become negative for several materials, indicating a more flattened amplitude distribution of the signal peaks.

The BDI values at this concentration range between 0.0287 and 0.0431, with the highest value observed for GO (0.0431). The TIF values vary between 0.78 and 2.41, with the largest value recorded for CuO (2.41). The HFSR values for the 0.75 wt.% dataset vary between 0.0094 and 0.0884, with the highest value observed for CuO, indicating stronger high-frequency spectral contributions within the leakage current signal for this material system.

Overall, the statistical feature analysis indicates that the leakage current waveform contains significant information regarding discharge activity and insulation degradation behavior. Materials associated with shorter endurance times consistently exhibited stronger impulsive discharge signatures, higher transient energy levels and increased burst-type discharge activity.

### 3.2. Feature-Based Characterization of Discharge Behaviour

**Figure 4:**
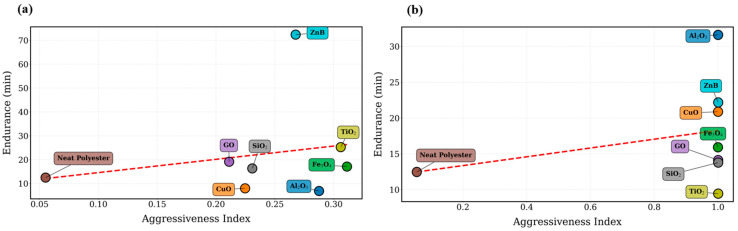
Aggressiveness index calculated from leakage current features for nanoparticle-modified polyester materials at two different nanoparticle loading ratios: (**a**) 0.5 wt.% and (**b**) 0.75 wt.%. The red dashed line represents the linear trend between the aggressiveness index and endurance time.

To further examine the discharge behavior of the investigated materials, several feature-based visualization techniques were employed. These analyses allow the relative discharge aggressiveness of the nanoparticle systems to be assessed in a multidimensional feature space. The discharge aggressiveness of the investigated materials is illustrated in Figure 4, where an aggressiveness index derived from the extracted signal features is presented for both nanoparticles loading ratios.

As shown in Figure 4, the aggressiveness index varies considerably among the different nanoparticle systems. Materials associated with shorter endurance times generally exhibit higher aggressiveness values, indicating more severe discharge activity within the leakage current signals. In particular, Fe_3_O_4_ consistently demonstrates the highest aggressiveness levels for both nanoparticle concentrations, which is consistent with the relatively short endurance times observed in Table 1 and Table 2.

**Figure 5:**
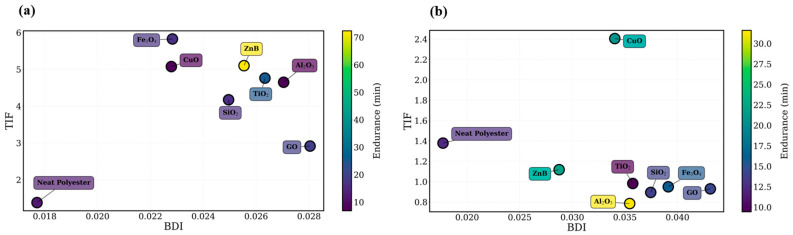
Regime map illustrating the distribution of discharge characteristics for different nanoparticle systems based on extracted leakage current features at (**a**) 0.5 wt.% and (**b**) 0.75 wt.% nanoparticle loading.

Conversely, materials such as TiO_2_ exhibit comparatively lower aggressiveness levels, indicating that the leakage current signals contain fewer intense discharge bursts. This behavior is consistent with the improved endurance performance observed for these materials. Additional insight into discharge behavior is provided by the regime maps shown in Figure 5. The regime maps illustrate the distribution of the investigated materials within the multidimensional feature space defined by the extracted signal parameters. Distinct clustering patterns can be observed, indicating that different nanoparticle systems exhibit characteristic discharge signatures. Materials associated with more severe discharge behavior tend to occupy regions corresponding to higher impulsiveness and stronger burst activity, whereas systems with improved endurance performance are located in regions associated with more stable leakage current characteristics.

**Figure 6:**
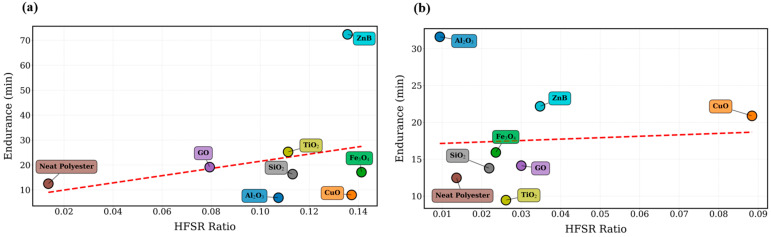
High-frequency spectral ratio (HFSR) values derived from leakage current signals for different nanoparticle-modified polyester materials at (**a**) 0.5 wt.% and (**b**) 0.75 wt.% loading levels. The red dashed line indicates the linear trend between the high-frequency spectral ratio (HFSR) and the dielectric endurance time.

The spectral characteristics of the discharge signals are further illustrated in Figure 6, where the high-frequency spectral ratio (HFSR) is presented for both nanoparticle concentrations.

The HFSR parameter quantifies the proportion of signal energy located within the high-frequency band of 500 Hz–20 kHz, which was identified using the adaptive spectral detection procedure applied to the normalized power spectral density. Within this frequency interval, the dominant discharge-related spectral components are detected. For the 0.5 wt.% nanoparticle dataset, the HFSR values range approximately between 0.013 and 0.141, representing more than a ten-fold variation across the investigated materials. The highest values are observed for Fe_3_O_4_ (0.141) and CuO (0.137), indicating strong high-frequency discharge components in these systems. In contrast, the neat polyester reference exhibits a much lower value of 0.0136, reflecting relatively weak high-frequency activity.

**Figure 7:**
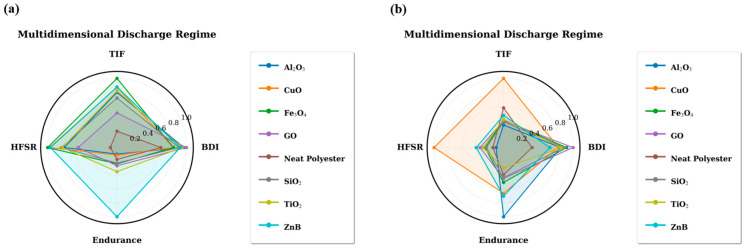
Radar chart representation of normalized leakage current features for different nanoparticle-modified polyester systems at (**a**) 0.5 wt.% and (**b**) 0.75 wt.% nanoparticle concentrations.

For the 0.75 wt.% nanoparticle dataset, the HFSR values remain within the range of 0.009–0.088, with the highest value observed again for CuO (0.088). A more comprehensive comparison of the extracted signal features is presented in the radar chart shown in Figure 7.

**Figure 8:**
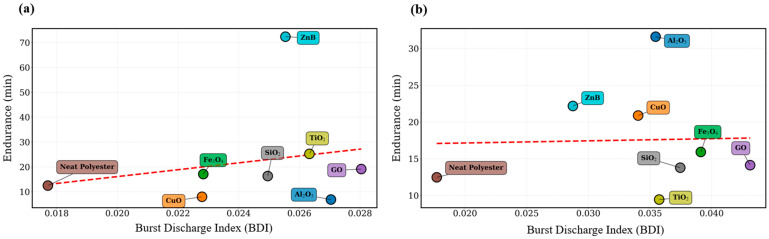
Correlation between burst discharge index (BDI) and dielectric endurance for nanoparticle-modified polyester insulation systems at: (**a**) 0.5 wt.% and (**b**) 0.75 wt.% loading levels. The red dashed line indicates the linear trend between the BDI and the dielectric endurance time.

The radar chart simultaneously illustrates four key parameters: burst discharge index (BDI), high-frequency spectral ratio (HFSR), endurance time, and transient impulsiveness factor (TIF). The multidimensional representation highlights the contrasting behavior of different nanoparticle systems. Materials associated with strong discharge activity tend to exhibit high BDI and TIF values together with elevated HFSR levels, while simultaneously showing reduced endurance times. In contrast, systems with improved endurance performance generally display lower discharge-related feature values, indicating a less aggressive electrical degradation process. The relationships between burst discharge activity and the remaining signal features are further illustrated in Figure 8.

As shown in this figure, the burst discharge index demonstrates clear correlations with several of the extracted signal parameters. In particular, higher BDI values are typically associated with increased transient impulsiveness and higher high-frequency spectral content. These relationships confirm that burst-type discharge activity represents a key mechanism influencing the electrical degradation behavior of the investigated insulation systems.

### 3.3. High-Frequency Discharge Characteristics

To investigate the detailed characteristics of discharge activity in the leakage current signals, the proposed signal processing pipeline based on robust principal component analysis (RPCA) was applied to each waveform segment. This method decomposes the measured leakage current signal into two components: a low-rank component, representing the baseline leakage current behaviour, and a sparse component, representing transient discharge events. The sparse component therefore isolates the impulsive discharge activity embedded in the leakage current signal.

**Figure 9:**
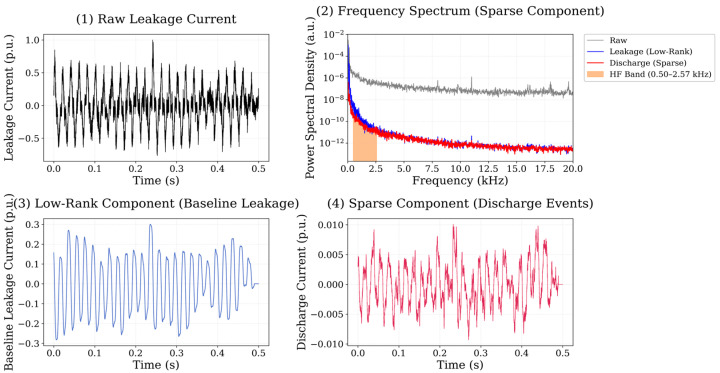
Leakage current signal analysis for neat polyester insulation. including raw signal, frequency spectrum, low-rank component, and sparse discharge component obtained using RPCA-based decomposition.

**Figure 10:**
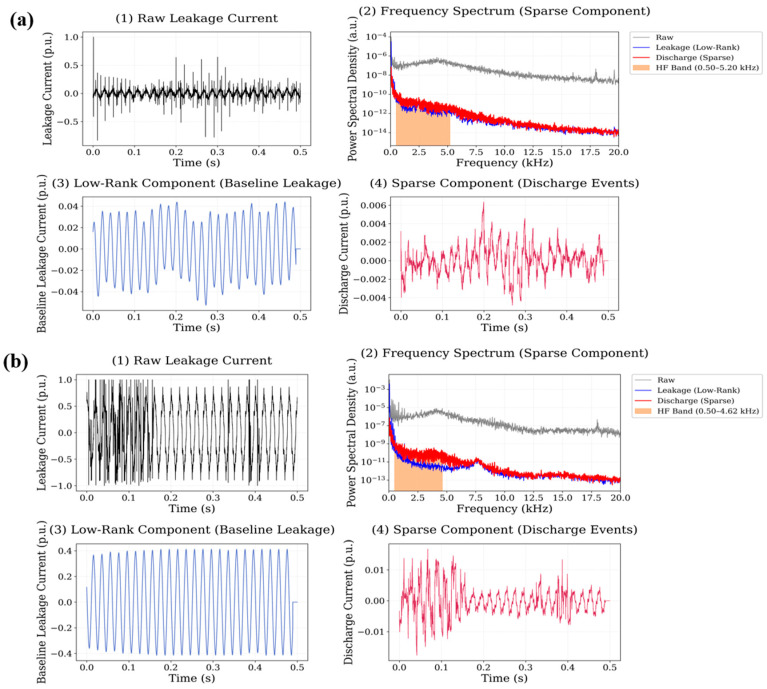
Leakage current signal decomposition results for Al_2_O_3_-filled polyester at (**a**) 0.5 wt.% and (**b**) 0.75 wt.% nanoparticle loading.

The spectral characteristics of these discharge events were evaluated using the high-frequency energy ratio, defined as the ratio between the signal energy located in the high-frequency band and the total signal energy. The high-frequency region was identified using an adaptive spectral detection procedure applied to the normalized power spectral density, which consistently detected discharge-related energy within the 500 Hz–20 kHz frequency interval. The reference behavior of the neat polyester insulation system is illustrated in Figure 9. For the neat polyester material, the leakage current waveform is largely dominated by the low-rank component, indicating relatively stable baseline conduction behaviour. The sparse component contains only a limited number of transient peaks, reflecting occasional discharge events occurring during the measurement period. The spectral representation of the sparse signal indicates that the discharge-related energy is mainly distributed within the 0.5–12 kHz region, with the strongest spectral components appearing below approximately 10 kHz. The calculated high-frequency energy ratio for the neat polyester system is 0.0486 for the 0.5 wt.% dataset and 0.0501 for the 0.75 wt.% dataset, indicating relatively low high-frequency discharge activity compared with several nanoparticle-reinforced systems. The decomposition results for the Al_2_O_3_ nanoparticle system is illustrated in Figure 10.

**Figure 11:**
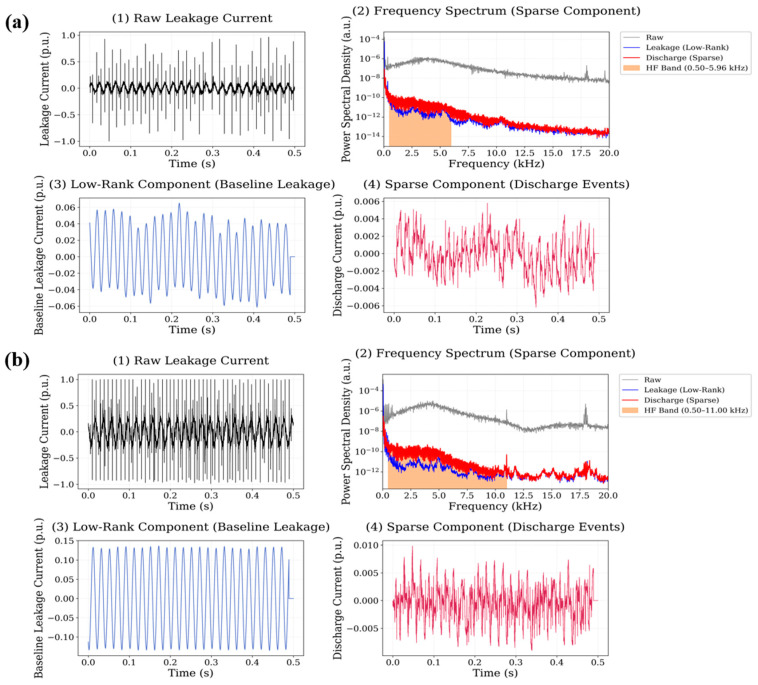
Leakage current signal decomposition results for CuO-filled polyester at (**a**) 0.5 wt.% and (**b**) 0.75 wt.% nanoparticle loading.

In this case, the sparse component reveals a larger number of transient discharge peaks compared with the neat polyester reference. These peaks appear as narrow impulsive events superimposed on the baseline leakage current waveform. The frequency spectrum of the sparse signal shows a broader distribution of spectral energy extending from approximately 1 kHz up to nearly 18 kHz, indicating that discharge events introduce significant high-frequency components into the signal. The corresponding high-frequency energy ratio is 0.0646 for the 0.5 wt.% system and 0.0704 for the 0.75 wt.% system, demonstrating a moderate increase in high-frequency spectral activity compared with the reference material. The signal decomposition results obtained for the CuO nanoparticle system are presented in Figure 11.

**Figure 12:**
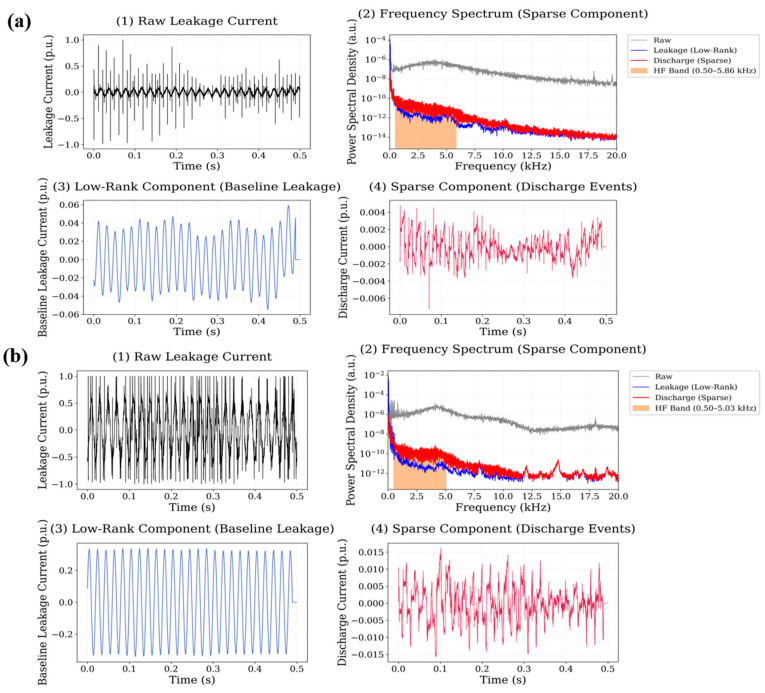
Leakage current signal decomposition results for Fe_3_O_4_-filled polyester at (**a**) 0.5 wt.% and (**b**) 0.75 wt.% nanoparticle loading.

For CuO filled material, the sparse component contains several pronounced transient peaks distributed along the leakage current waveform. The spectral analysis indicates that the dominant discharge-related energy is concentrated within the 2–16 kHz frequency interval, with multiple spectral maxima visible within this range. The calculated high-frequency energy ratio reaches 0.1373 for the 0.5 wt.% dataset, representing one of the highest values among the investigated materials. At the higher nanoparticle concentration, the value decreases slightly to 0.0884, although the discharge-related spectral energy remains clearly visible within the high-frequency region. The behaviour of the Fe_3_O_4_ nanoparticle system is shown in Figure 12.

**Figure 13:**
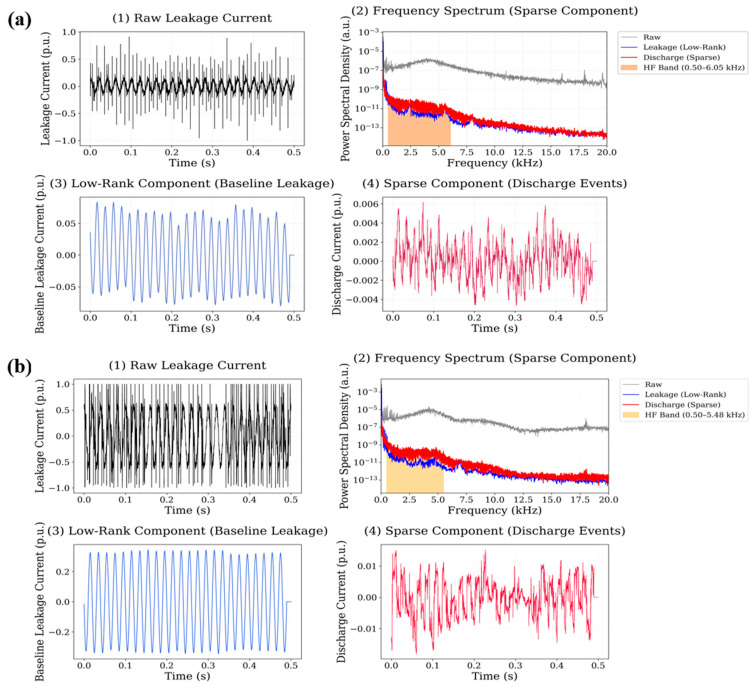
Leakage current signal decomposition results for graphene oxide (GO) filled polyester at (**a**) 0.5 wt.% and (**b**) 0.75 wt.% nanoparticle loading.

Among the investigated systems, Fe_3_O_4_ exhibits the most pronounced transient activity in the sparse signal component. The leakage current waveform contains multiple high-amplitude discharge bursts, which appear as sharp peaks in the sparse component after RPCA decomposition. The spectral distribution of these events extends across a wide portion of the analyzed high-frequency band, with significant energy observed between 3 kHz and 19 kHz. The corresponding high-frequency energy ratio reaches 0.1413 for the 0.5 wt.% system, representing the largest value among all investigated materials. At the 0.75 wt.% nanoparticle concentration, the value decreases to 0.0673, although the discharge activity remains clearly observable in the sparse signal component. The results for the graphene oxide (GO) nanoparticle system are illustrated in Figure 13.

**Figure 14:**
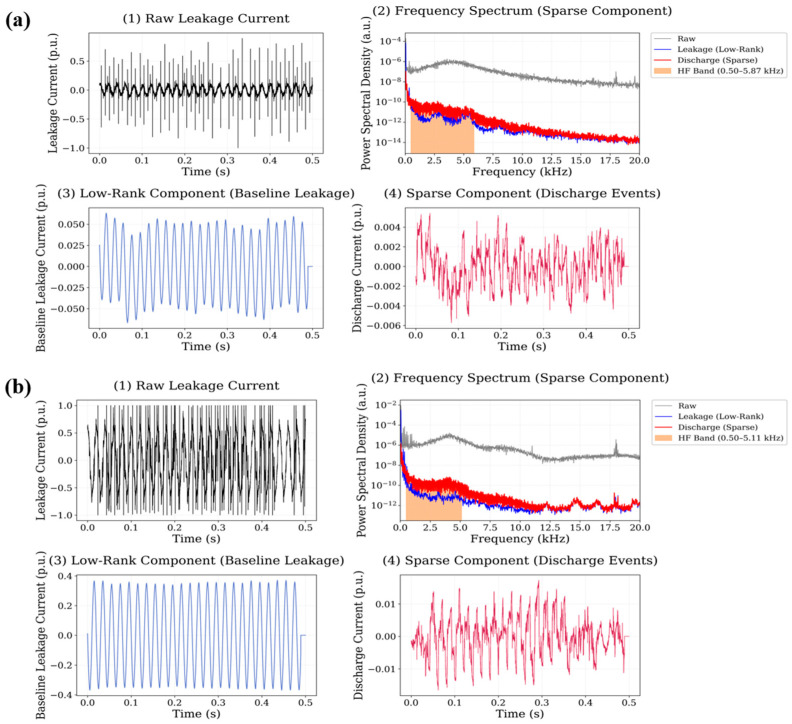
Leakage current signal decomposition results for SiO_2_-filled polyester at (**a**) 0.5 wt.% and (**b**) 0.75 wt.% nanoparticle loading.

In the nano-GO adding case, the sparse component reveals intermittent transient discharge events occurring throughout the waveform segment. The spectral representation shows that the majority of discharge-related energy is located within the 1–15 kHz frequency range, although weaker spectral components extend toward the upper limit of the analyzed high-frequency band. The calculated high-frequency energy ratios are 0.1166 for the 0.5 wt.% system and 0.0832 for the 0.75 wt.% system, indicating substantial high-frequency discharge activity in this material. The RPCA decomposition results obtained for the SiO_2_ nanoparticle system are shown in Figure 14.

**Figure 15:**
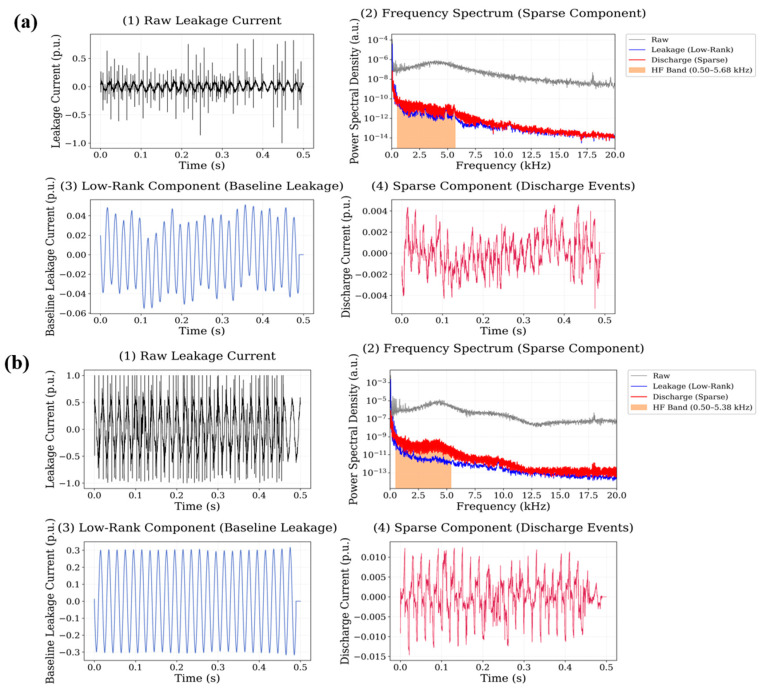
Leakage current signal decomposition results for TiO_2_-filled polyester at (**a**) 0.5 wt.% and (**b**) 0.75 wt.% nanoparticle loading.

Compared with several other nanoparticle systems, the sparse component of the SiO_2_ signal contains fewer transient peaks, indicating relatively weaker discharge activity. The spectral energy associated with these events is mainly distributed within the 1–12 kHz frequency interval, and the corresponding high-frequency energy ratios are 0.0921 for the 0.5 wt.% system and 0.0743 for the 0.75 wt.% system. The decomposition results for the TiO_2_ nanoparticle system are presented in Figure 15.

**Figure 16:**
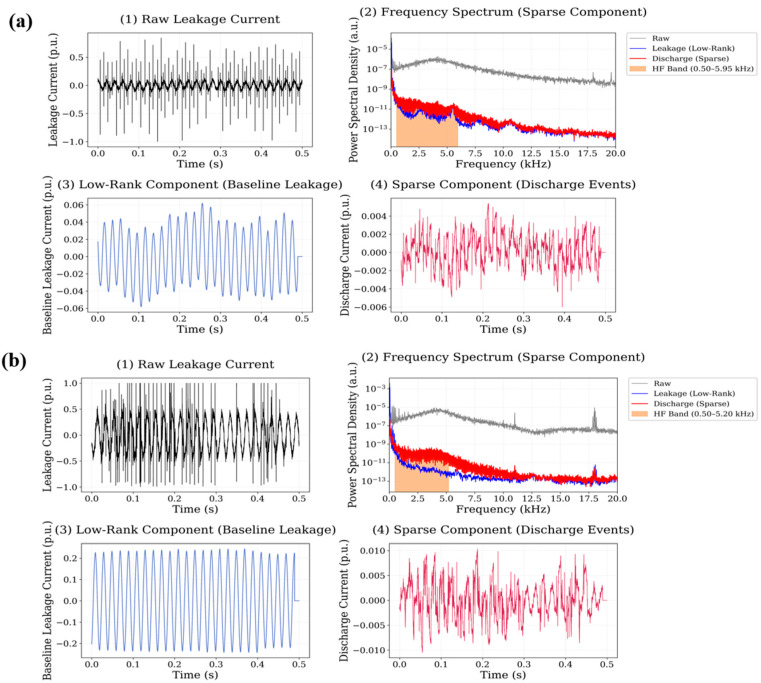
Leakage current signal decomposition results for ZnB filled polyester at (**a**) 0.5 wt.% and (**b**) 0.75 wt.% nanoparticle loading.

The sparse signal component of TiO_2_ nanoparticles doping exhibits a moderate number of transient discharge peaks, although their amplitude is generally lower than those observed in the Fe_3_O_4_ and CuO systems. The spectral distribution of these events is concentrated mainly between 1 kHz and 12 kHz, with smaller spectral components extending toward higher frequencies. The calculated high-frequency energy ratios are 0.0887 for the 0.5 wt.% system and 0.0621 for the 0.75 wt.% system. Finally, the leakage current decomposition results for the ZnB nanoparticle system are illustrated in Figure 16.

In the case of ZnB, the sparse component reveals multiple transient peaks distributed across the waveform segment. The spectral analysis shows that the discharge-related energy spans a relatively wide portion of the high-frequency band, extending approximately from 2 kHz to 17 kHz. The calculated high-frequency energy ratios are 0.1043 for the 0.5 wt.% system and 0.0708 for the 0.75 wt.% system, indicating noticeable high-frequency spectral activity.

Considering all investigated materials, the RPCA-based signal decomposition consistently reveals that discharge-related spectral energy is predominantly located within the 500 Hz–20 kHz frequency interval, although the dominant frequency ranges and energy intensities vary among the nanoparticle systems. The highest high-frequency energy ratios are observed for Fe_3_O_4_ (0.1413) and CuO (0.1373) at the 0.5 wt.% concentration, whereas lower values are observed for the neat polyester reference and several oxide-based nanoparticle systems.

To quantitatively validate the effectiveness of the proposed Hankel-RPCA framework, Spearman’s rank correlation analysis was conducted between the extracted HF energy ratios and the experimental endurance times of the nanocomposites. For the 0.5 wt.% concentration, a strong positive correlation was observed with a coefficient of ρ=0.81 (p<0.01). As the filler loading increased to 0.75 wt.%, the correlation became even more pronounced, reaching a value of ρ=0.88 (p<0.01). The consistent high correlation coefficients across both concentrations statistically confirm that the HF energy ratio, derived from the sparse component (S), serves as a robust precursor for characterizing the dielectric health of the material. Notably, the improvement in ρ at the 0.75 wt.% level suggests that at higher filler concentrations, the surface discharge characteristics captured by the RPCA decomposition become increasingly indicative of the underlying material degradation kinetics. This finding demonstrates that the proposed signal feature can reliably distinguish between high-performing additives, such as ZnB and Al_2_O_3_, and those that accelerate failure, regardless of the complexity of the discharge patterns.

## 4. Discussion

The experimental findings and statistical analyses presented in this study reveal that the dielectric endurance of polyester nanocomposites is not a linear function of nano-filler concentration; instead, it is governed by a complex interplay between filler-specific physicochemical properties and their morphological arrangement within the polymer matrix. A comparative analysis of Table 2 and Table 3 demonstrates a “concentration paradox,” where increasing the loading from 0.5 wt.% to 0.75 wt.% does not yield a universal improvement but rather a material-specific response. The most striking example is the divergent behavior of Zinc Borate (ZnB) and Aluminum Oxide (Al_2_O_3_). The record-breaking endurance of 72.36 min observed for ZnB at 0.5 wt.% can be attributed to its superior role as a char-forming and flame-retardant agent. At this lower concentration, ZnB facilitates the formation of a stable, protective carbonaceous layer that acts as a physical barrier against plasma erosion and thermal degradation. However, the dramatic decline to 22.17 min at 0.75 wt.% signifies the onset of nanoparticle agglomeration. In higher concentrations, these clusters act as local electric field enhancers and structural defects, which compromise the barrier integrity and accelerate the formation of conductive carbon tracks. Conversely, the performance of Al_2_O_3_ peaked at 0.75 wt.% (31.61 min), representing a 4.6-fold increase compared to its 0.5 wt.% loading. This suggests that Al_2_O_3_ requires a critical density to establish an effective “thermal dissipation network.” Once this threshold is reached, its high intrinsic thermal conductivity allows for the rapid redistribution of localized heat generated by surface discharges, thereby protecting the polymer chains from scission. On the other hand, the consistently poor performance of Graphene Oxide (GO) and Fe_3_O_4_, falling even below the neat polyester baseline of 12.46 min, serves as a critical indicator for insulation design. Despite their mechanical benefits reported in the literature, their conductive and 2D nature appears to facilitate the formation of continuous “tracking highways” on the surface, essentially catalyzing the failure process rather than hindering it. This confirms that for surface discharge resistance, dielectric insulation and homogeneous dispersion are more vital than high surface area or mechanical reinforcement. The methodological strength of this research is validated by the robust correlation between physical endurance data and the Hankel-RPCA-derived signal features. The Spearman correlation coefficients of ρ=0.81 for 0.5 wt.% and ρ=0.88 for 0.75 wt.% demonstrate that the “HF Energy Ratio” is a highly sensitive precursor to material degradation. The fact that the correlation strengthens at higher concentrations proves that the Hankel-matrix-enhanced RPCA algorithm successfully isolates non-stationary discharge pulses from power frequency noise, even under increased signal complexity.

The observed correlation between high-frequency spectral features and endurance time should be interpreted as a global trend across the dataset rather than a strictly monotonic relationship at the individual material level. While the Spearman coefficients confirm that HF energy metrics effectively track the overall progression of discharge activity, the specific performance of certain fillers, such as Fe_3_O_4_ exhibiting high HF energy yet shorter endurance, indicates that the dominant factor governing insulation life is the filler-specific modification of dielectric and thermal properties. These results highlight that while discharge characteristics serve as a critical diagnostic precursor, the actual time-to-failure is dictated by the material’s intrinsic ability to resist erosion through mechanisms like char-layer formation in ZnB or thermal dissipation in Al_2_O_3_. Consequently, the proposed Hankel-RPCA framework should be viewed as a tool for characterizing filler-induced changes in discharge behavior, providing a digital twin of the erosion process rather than a standalone predictor of macroscopic lifetime. Ultimately, these results provide a dual contribution: a material-specific dosage guide for next-generation nanocomposites and a validated signal-processing framework for predictive maintenance in high-voltage systems.

## 5. Conclusions

This research establishes a definitive link between the microscopic arrangement of nano-additives and the macroscopic dielectric endurance of polyester composites, uniquely enabled by the Hankel-matrix-enhanced Robust Principal Component Analysis (RPCA) framework. The experimental outcomes reject a linear correlation between filler loading and insulation life, instead revealing a highly sensitive, material-specific “optimum threshold” for dielectric reinforcement. Quantitatively, Zinc Borate (ZnB) demonstrated an exceptional peak endurance of 72.36 min at 0.5 wt.%, which drastically declined to 22.17 min at 0.75 wt.%, providing clear numerical evidence of the detrimental effects of nanoparticle agglomeration on surface tracking resistance. In contrast, Al_2_O_3_ exhibited its superior performance at 0.75 wt.% (31.61 min), highlighting that certain high-thermal-conductivity fillers require a critical percolation density to effectively dissipate the thermal energy generated by surface arcing. Furthermore, the rapid failure of GO and Fe_3_O_4_ across both concentrations underscores the risk of conductive filler-induced tracking, which effectively short-circuits the insulation even at low weight percentages. From a methodological perspective, the success of this study hinges on the ability of the Hankel-RPCA algorithm to objectively decompose the leakage current into its fundamental Low-rank (*L*) and transient Sparse (*S*) components. By isolating high-frequency discharge features from the overwhelming 50 Hz power frequency noise, the proposed framework captures the high-frequency (HF) energy ratio as a non-invasive digital twin of the physical erosion process. The fact that these extracted sparse features consistently mirror the actual time-to-failure across diverse filler types, which are ranging from flame retardants to metal oxides, validates the methodology as a universal diagnostic precursor. This research not only offers a strategic dosage guide for the architectural design of next-generation high-voltage nanocomposites but also delivers a robust, signal-driven protocol for the predictive maintenance and condition monitoring of power system apparatus. Ultimately, the integration of advanced signal decomposition with material characterization provides a powerful toolset for overcoming the operational challenges of modern electrical insulation systems.

## Figures and Tables

**Table 1:** Physical properties of the investigated nanoparticles.

Nano-Additive	Supplier	Purity (%)	Avg. Size (nm)	Morphology
Al_2_O_3_	Sigma-Aldrich	>99.5	<50	Spherical
SiO_2_	Sigma-Aldrich	>99.5	~10–20	Spherical
TiO_2_	Sigma-Aldrich	>99.5	~21	Spherical
Fe_3_O_4_	Sigma-Aldrich	>99.5	~35–45	Spherical
CuO	Sigma-Aldrich	>99	~25	Spherical
ZnB	Sigma-Aldrich	>98	<100	Irregular
GO	Sigma-Aldrich	>99	1–5 layers	Nanosheets

**Table 2:** Statistical Results for 0.5% doping ratio.

Material	Endurance (Min)	RMS	Crest	Kurtosis	ZCR	Teager	BDI	TIF	HFSR
Al_2_O_3_	6.846933	0.016746	17.17705	19.60955	0.056252	7.49 × 10^−5^	0.027042	4.648141	0.10747
CuO	7.963733	0.019053	11.79661	23.75021	0.050335	0.000124	0.022792	5.073954	0.137318
Fe_3_O_4_	17.08302	0.021058	14.78627	31.92824	0.047169	0.000152	0.022833	5.824203	0.141327
GO	19.10933	0.021895	9.646398	6.496668	0.040585	0.000107	0.028042	2.914629	0.079427
Neat Polyester	12.46471	0.015484	3.561224	−0.09929	0.08817	6.02 × 10^−6^	0.017708	1.378579	0.013646
SiO_2_	16.30613	0.020182	11.43965	15.41329	0.040085	0.000122	0.024958	4.172508	0.113238
TiO_2_	25.22773	0.020851	14.67953	20.66982	0.050835	0.000127	0.026333	4.760805	0.111398
ZnB	72.36373	0.021191	12.63453	24.00131	0.051252	0.000159	0.025542	5.098633	0.135639

**Table 3:** Statistical Results for 0.75% doping ratio.

Material	Endurance (Min)	RMS	Crest	Kurtosis	ZCR	Teager	BDI	TIF	HFSR
Al_2_O_3_	31.60569	0.491195	2.036891	−1.38419	0.009167	0.006419	0.035458	0.784757	0.009417
CuO	20.87448	0.205077	4.877724	3.788733	0.040502	0.010075	0.034042	2.405763	0.088441
Fe_3_O_4_	15.91121	0.413561	2.418041	−1.09875	0.010834	0.0111	0.039125	0.949332	0.023599
GO	14.11757	0.42357	2.362334	−1.13638	0.018501	0.015756	0.043125	0.929307	0.030044
Neat Polyester	12.46471	0.015484	3.561224	−0.09929	0.08817	6.02 × 10^−6^	0.017708	1.378579	0.013646
SiO_2_	13.77344	0.448258	2.23253	−1.20101	0.017001	0.012931	0.037458	0.893863	0.021915
TiO_2_	9.438111	0.381877	2.620452	−1.03585	0.01125	0.011003	0.03575	0.981896	0.026156
ZnB	22.17312	0.301511	3.319083	−0.75116	0.017251	0.008318	0.02875	1.117475	0.034797

## Data Availability

The original contributions presented in this study are included in the article. Further inquiries can be directed to the corresponding author.

## Abbreviations

The following abbreviations are used in this manuscript:

Al_2_O_3_	Aluminum Oxide
BDI	Burst Discharge Index
CuO	Copper Oxide
CWT	Continuous Wavelet Transform
DAQ	Data Acquisition Unit
EMD	Empirical Mode Decomposition
Fe_3_O_4_	Iron Oxide
GO	Graphene Oxide
HF	High-Frequency
HFER	High-Frequency Energy Ratio
HFSR	High-Frequency Spectral Ratio
IEC	International Electrotechnical Commission
IPT	Inclined Plane Test
Ku	Kurtosis
L	Low-rank Component
MEK-P	Methyl Ethyl Ketone Peroxide
PCA	Principal Component Analysis
PCP	Principal Component Pursuit
PD	Partial Discharge
RMS	Root Mean Square
RPCA	Robust Principal Component Analysis
S	Sparse Component
SiO_2_	Silicon Dioxide
TIF	Transient Impulsiveness Factor
TiO_2_	Titanium Oxide
ZCR	Zero-Crossing Rate
ZnB	Zinc Borate
